# Exploring the molecular mechanisms by which exercise improves cognitive impairment in Parkinson’s disease based on the brain-gut microbiota axis: bibliometrics and visualization analysis

**DOI:** 10.3389/fmicb.2026.1750505

**Published:** 2026-03-11

**Authors:** Wanhong Wang, Ting Ma, Zhengang Qiu, Xin Zhang, Wenyu Sun

**Affiliations:** 1 College of Rehabilitation Medicine, Shandong University of Traditional Chinese Medicine, Jinan, Shandong, China; 2 Department of Rehabilitation Physiotherapy, Affiliated Hospital of Shandong University of Traditional Chinese Medicine, Jinan, Shandong, China

**Keywords:** brain-gut microbiota axis, cognitive impairment, molecular mechanism, movement, neuroprotection, Parkinson’ s disease, visual analysis

## Abstract

**Background:**

Parkinson’ s disease (PD) is a common neurodegenerative disease, and its cognitive impairment, as an important non-motor symptom, seriously affects the quality of life of patients. In recent years, the brain-gut flora axis, as a key pathway connecting the central nervous system(CNS) and intestinal microecology, has gradually become the forefront of research on cognitive dysfunction in PD. A large number of studies have shown that the imbalance of intestinal flora is closely related to the occurrence of cognitive impairment in PD, revealing molecular mechanisms such as inflammatory response, neurotransmitter metabolism, and immune regulation. As a safe and effective non-drug intervention, exercise can regulate the structure of intestinal flora and its metabolites, activate relevant signaling pathways, exert a neuroprotective effects, and improve cognitive function. In view of the large increase in related research in recent years, this study aims to use bibliometric methods to systematically review the global research status and trends of exercise treatment for cognitive dysfunction in PD patients, focusing on analyzing the relevant mechanisms of how exercise intervention interferes with Parkinson’ s cognitive ability by affecting the intestinal microecology. To explore the synergistic effect of exercise and intestinal flora regulation and its potential therapeutic value. This review aims to provide a new perspective on the pathological mechanism of cognitive impairment in PD and provide scientific basis for the optimization of exercise intervention strategies.

**Methods:**

Literature related to exercise and cognitive dysfunction in PD published between 1999 and 2024 was searched in the Science Citation Index Expanded (SCIE) and Social Science Citation Index (SSCI) databases based on the Web of Science Core Collection (WoSCC). Bibliometric analysis was performed using VOSviewer and CiteSpace software to analyze data on countries, institutions, authors, journals, keywords, citations, and to generate visual maps.

**Results:**

Among 332 publications, annual output peaked in 2022. The USA led in publications (*n* = 117). Key institutions included Eberhard Karls University of Tübingen. Research hotspots centered on mild cognitive impairment, dementia, and aerobic exercise. Emerging trends include home-based interventions and basal ganglia mechanisms.

**Conclusion:**

This study aims to use bibliometric methods to systematically review the global research status and trends of exercise treatment for cognitive dysfunction in PD patients, focusing on analyzing how exercise intervention affects the intestinal microecology and its interaction with the central CNS, exploring the synergistic effect of exercise and intestinal flora regulation and its potential therapeutic value, and providing a new perspective and path for understanding the intervention of cognitive impairment in PD.

## 1 Introduction

PD is the second most common neurodegenerative disease. In addition to the classic movement disorder symptoms, its cognitive impairment, as an important non-motor symptom, has attracted increasing attention in the clinical and research fields. Cognitive impairment not only significantly affects patients’ quality of life and social functions, but is also closely related to the prognosis of the disease (Armstrong and Okun, 2020). Currently, there is no clear disease-modifying therapy for cognitive impairment in PD, and existing central acetylcholinesterase inhibitors and NMDA receptor antagonists can only temporarilyh relieve dementia symptoms (Annesi, 2019). Therefore, exploring new intervention strategies, especially non-drug treatments, has become an urgent issue to be solved. In recent years, the brain-gut microbiota axis-a bidirectional regulatory pathway connecting the CNS and intestinal microecology-has emerged as a core focus in PD cognitive dysfunction research. This axis communicates through three key pathways: (1) Neural pathway: Intestinal flora and their metabolites (e.g.,short chain fatty acids, SCFAs) regulate vagus nerve activity to modulate CNS function; (2) Immune pathway: Intestinal flora imbalance disrupts intestinal barrier integrity, allowing bacterial endotoxins (e.g., lipopolysaccharide, LPS) to enter the bloodstream, activate systemic inflammation, and cross the blood–brain barrier (BBB) to induce neuroinflammation; (3) Endocrine pathway: Flora metabolites influence the synthesis and release of neurotransmitters (e.g., 5-hydroxytryptamine, dopamine) and neurotrophic factors, directly affecting neuronal survival and synaptic plasticity (Sun and Armstrong, 2021). Clinical studies confirm that PD patients exhibit reduced intestinal flora diversity, with decreased Firmicutes and Bifidobacteria (beneficial flora) and increased Proteobacteria (pro-inflammatory flora), and this flora composition is significantly correlated with cognitive function scores (Schaeffer et al., 2019). Specifically, gut microbiota-related inflammation and BBB permeability changes promote abnormal protein aggregation (e.g., alpha-synuclein) and neuronal damage, becoming key drivers of cognitive impairment (Gonzalez-Latapi et al., 2021).

As a safe and effective non-drug intervention, exercise has been consistently shown to improve cognitive and motor function in PD patients (Dirnberger and Jahanshahi, 2013). The link between exercise and the brain-gut microbiota axis is multi-faceted: exercise first regulates intestinal flora structure by increasing beneficial flora abundance (e.g., Akkermansia, Lachnospiraceae) and restoring flora diversity (Feng et al., 2020); second, exercise enhances intestinal barrier function, reducing LPS leakage and systemic inflammation (Murray et al., 2014); finally, exercise-induced changes in flora metabolites (e.g., increased SCFAs) penetrate the BBB to regulate neuroinflammation, promote neurotrophic factor (e.g., BDNF) expression, and inhibit oxidative damage (Chen and Song, 2019). These mechanisms collectively underpin exercise’s neuroprotective effects, but their global research trends and quantitative relationships remain to be systematically summarized via bibliometric analysis.

By conducting bibliometric analysis in this field, the development trends and dynamics of a certain field can be intuitively displayed, reflecting current research hotspots and cutting-edge directions (Van Eck and Waltman, 2017). In this study, based on the Web of science core collection database, CiteSpace software was used to analyze related research on exercise in cognitive impairment in PD patients, and draw a visual map to reveal the development process, research hot spots and development trends in this field. Focusing on analyzing how exercise intervention affects the intestinal microecology and its interaction with the CNS, combined with the latest animal models and clinical research, we explore the synergistic effect of exercise and intestinal flora regulation and its potential therapeutic value, and provide a reference for the research and application of exercise in the treatment of cognitive impairment in PD patients.

## 2 Methodology

### 2.1 Data sources

**Figure 1:**
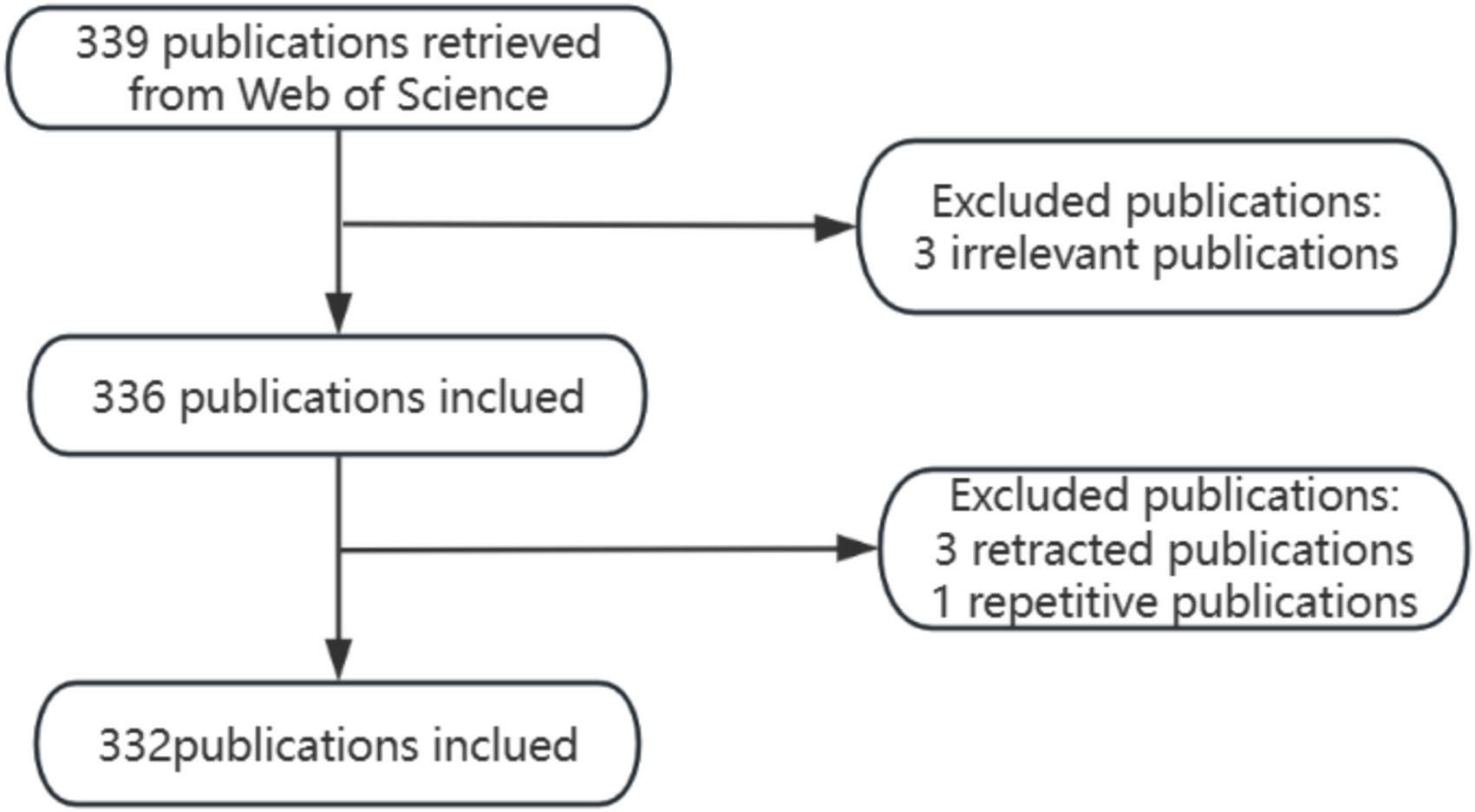
Data retrieval and analysis process.

This study was based on the Web of Science core collection database, and the literature was screened from Science Citation Index Expanded (SCI-EXPANDED)--1999-present, Social Sciences Citation Index (SSCI)--1999--2021 two core databases, and the search terms were TS = (“physical activity” OR “exercise” OR “aerobic exercise” OR “resistance exercise” OR “endurance exercise” OR “high-intensity exercise”) AND TS = (“parkinson disease” OR” Idiopathic Parkinson’s Disease “OR “Lewy Body Parkinson’ s Disease” OR “Parkinson’ s Disease, Idiopathic” OR “Parkinson’ s Disease. Idiopathic” OR ‘Parkinson’ s Disease, Lewy Body’ OR ‘Parkinson’s Disease, Idiopathic’ OR ‘Parkinson’ s Disease, Lewy Body’ OR” Parkinson’ s Disease, Lewy Body Idiopathic” OR ‘Parkinson’s Disease’ OR” Idiopathic Parkinson Disease “OR “Lewy Body Parkinson Disease” OR “Primary Parkinsonism” OR” Parkinsonism, Primary“) AND TS = (“cognitive dysfunction” OR” cognitive impairment* “OR “neurocognitive disorder*” OR “cognitive decline”). The search period was set from January 1999 to January 2025, and the language was set to English. Initially, 339 articles were searched in the literature, and the article types were limited to Article and Review, and 7 articles that were not related to the research topic were removed by reading the titles and abstracts of the articles. After the above screening process, 332 documents were finally included, from which data such as country, institution, author, journal, keywords and citation information were extracted for bibliometric analysis, as shown in Figure 1.

### 2.2 Research tools

In this study, we mainly used VOSviewer and Citespace software for bibliometric analysis. VOSviewer, as a scientific mapping software, was developed by Van Eck and Waltman’s research team at Leiden University, the Netherlands, in 2009 (Van Eck and Waltman, 2017). In this study, VOSviewer (V1.6.19) software was used to visualize and analyze countries, institutions, authors, keywords, etc. CiteSpace was developed by Dr. Chao-Mei Chen and his team in 2004, through which trends within the field can be presented (Van Eck and Waltman, 2017). Keyword clustering analysis, keyword emergence, and journal biplot overlay analysis were performed using CiteSpace (V.6.3. R1).

## 3 Results

### 3.1 Number of annual publications

**Figure 2:**
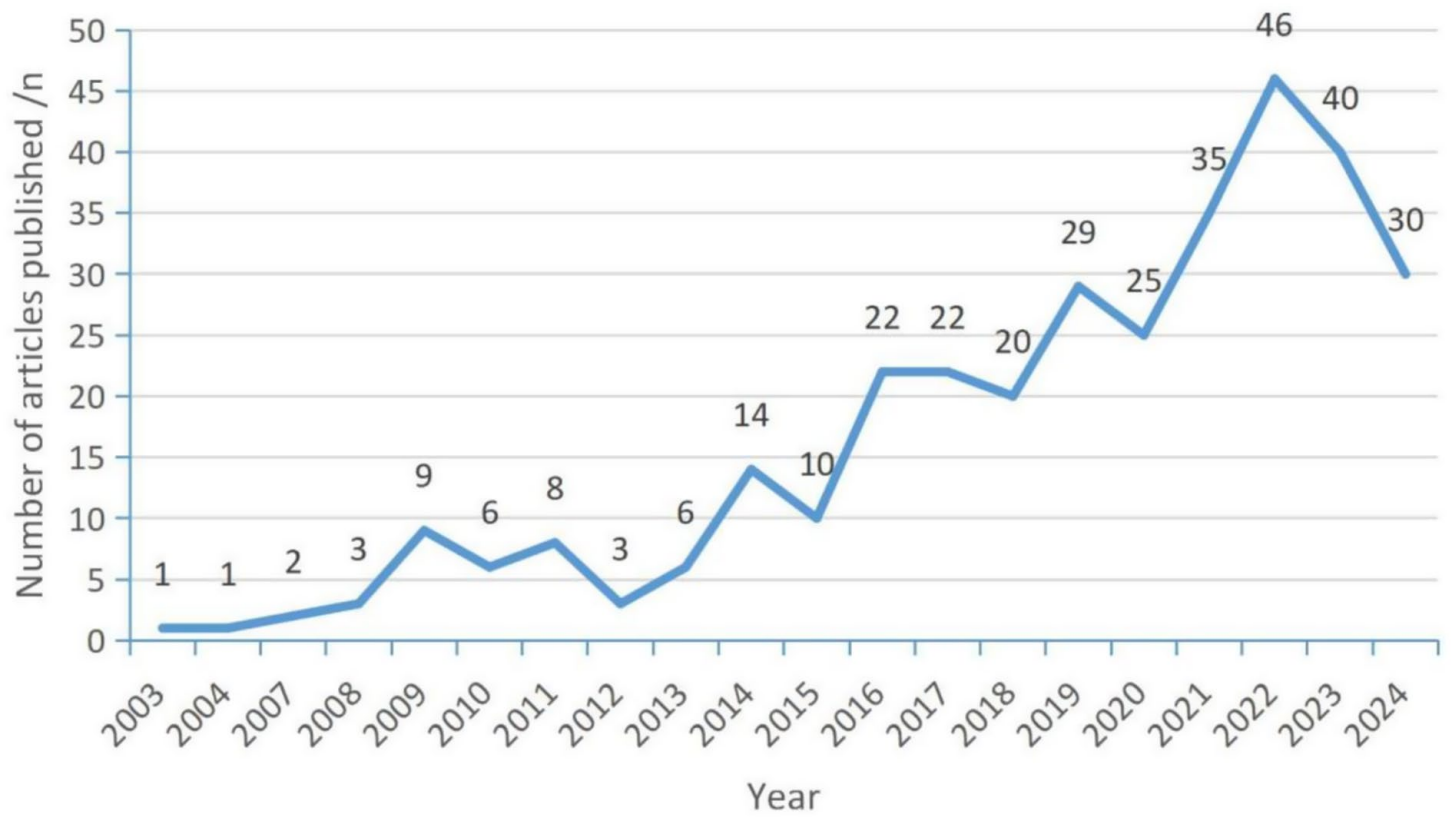
Number of annual publications from 1999 to 2024.

After screening, a total of 332 publications related to the study topic were identified. Figure 2 shows the number and trend of annual publications from 2003 to 2024. From a general point of view, the number of publications shows an increasing trend, and the growth rate is faster in recent years with more publications, and the number of publications peaks in 2022, and the number of publications decreases in the last 2 years.

### 3.2 Countries/regions

**Figure 3:**
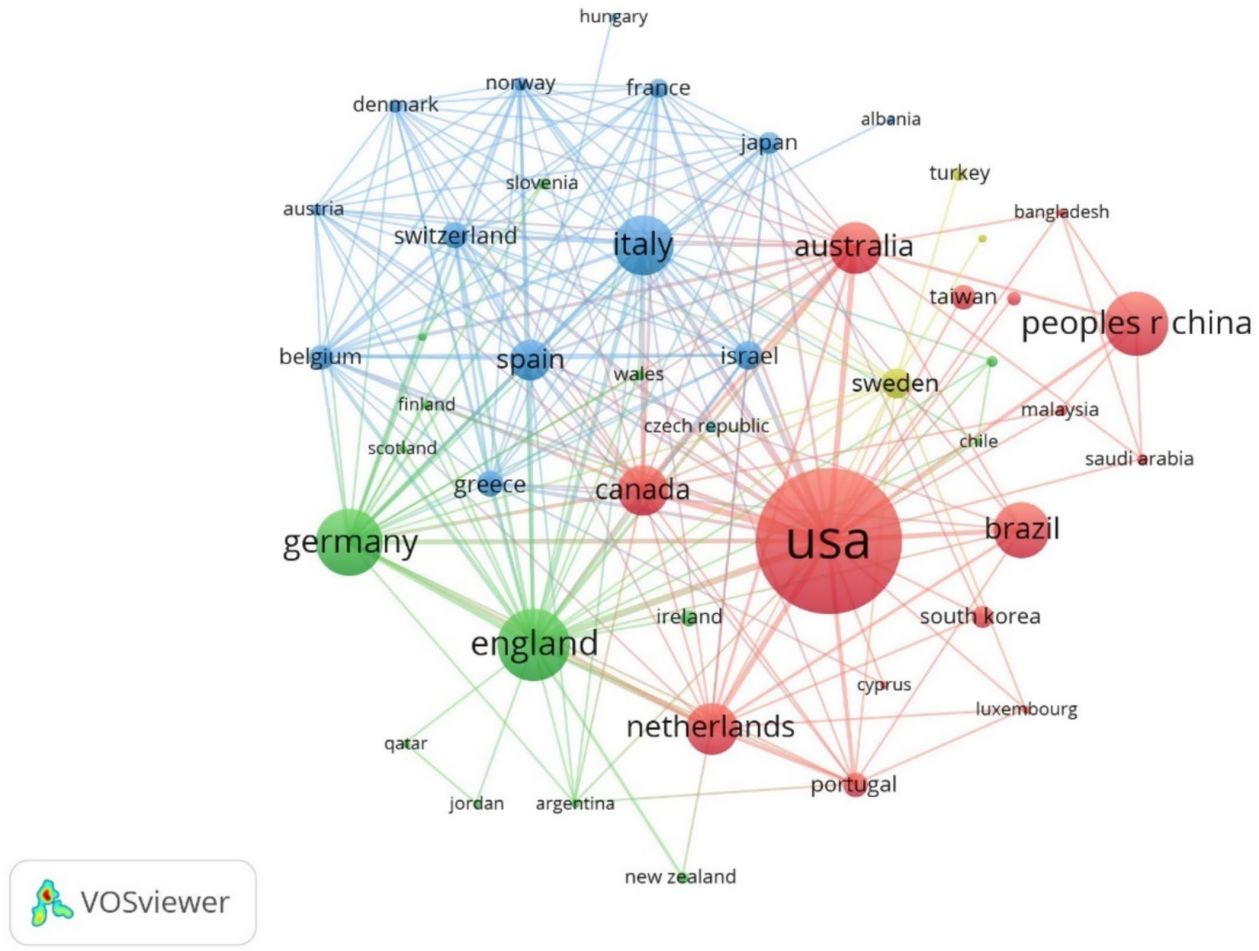
Cluster-based countries/regions collaboration map.

**Figure 4:**
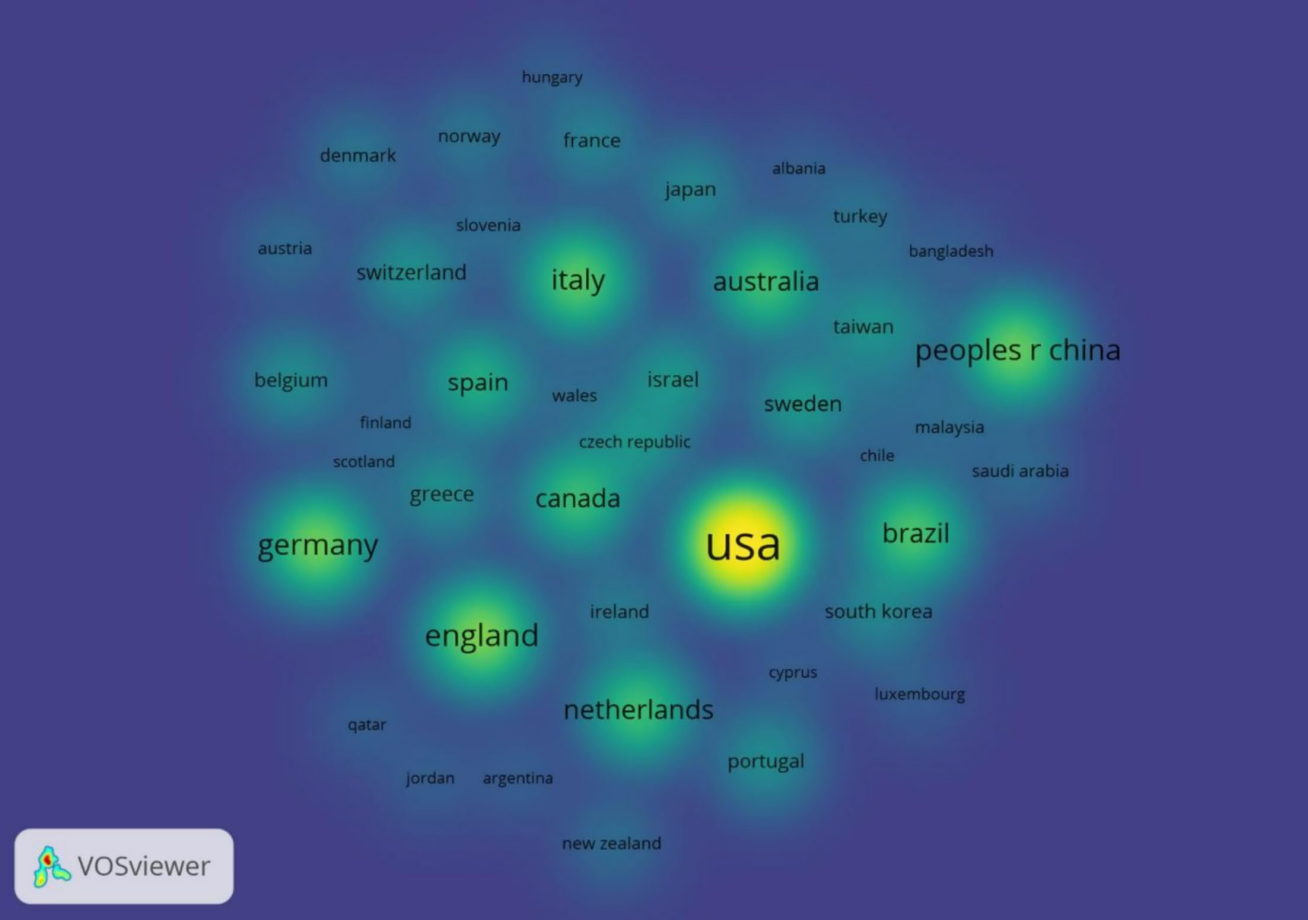
Countries/regions density map.

The analysis results show that the country with the largest number of articles is the United States, with a total of 117 articles, followed by the United Kingdom and Germany, with 38 and 34 articles respectively, and the detailed information of the top 5 countries/regions in terms of the number of articles is shown in Table 1. From the centrality point of view, the centrality of Germany, the UK, and Italy is higher, respectively 0.47, 0.3, and 0.27, which indicates that that these countries maintain close cooperation with other countries/regions, as shown in Figure 3. From the heat map of publication, the United States has a higher research heat in this field and has achieved certain research results in this field, as shown in Figure 4.

**Table 1:** Top 10 countries/regions in terms of the number of publications.

Rangking	Country/region	Publications	Centrality
1	United States	117	0.2
2	England	38	0.3
3	Germany	34	0.47
4	People’s Republic of China	32	0
5	Italy	29	0.27

### 3.3 Institutions

**Figure 5:**
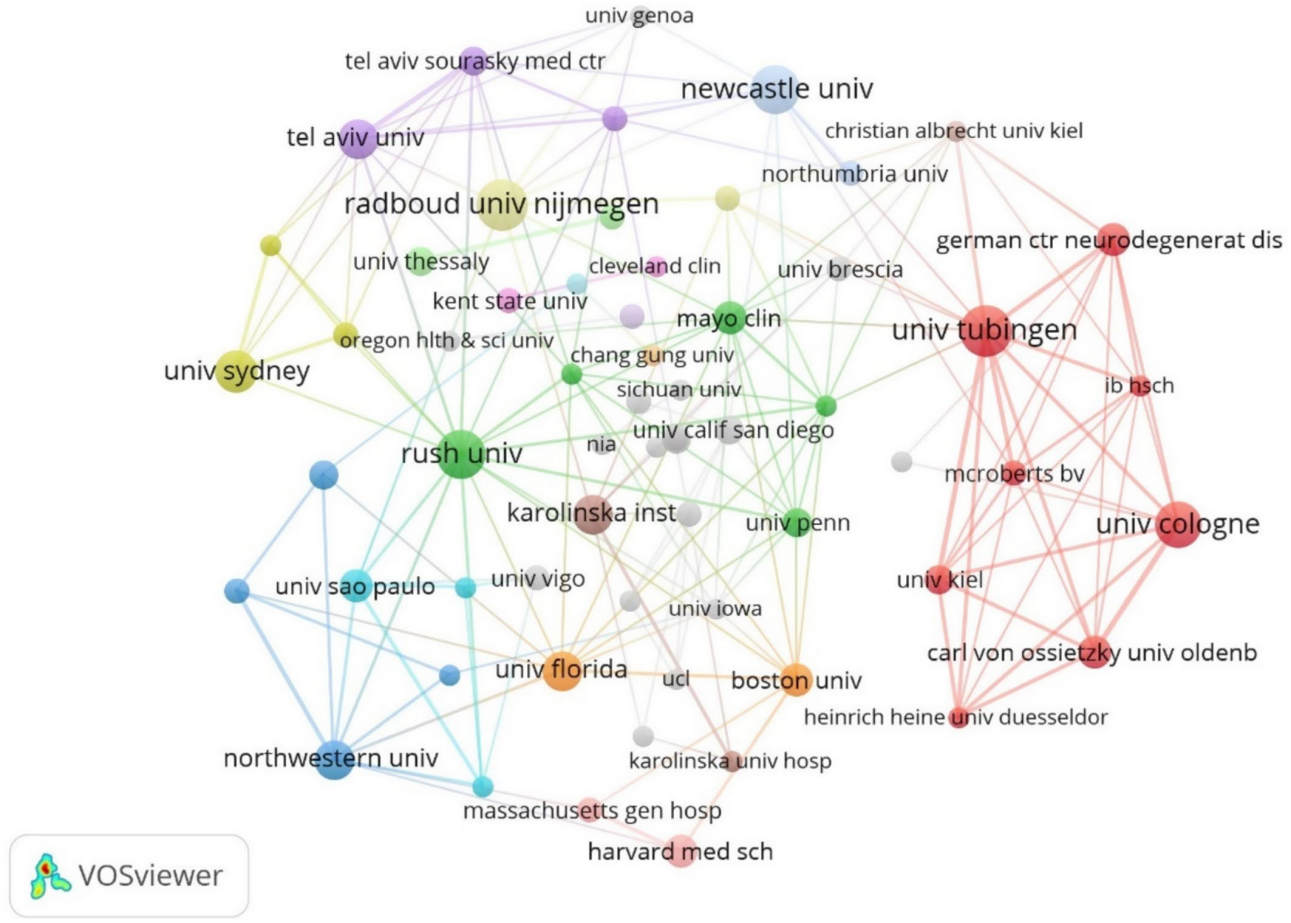
Cluster-based institutional collaboration map.

**Figure 6:**
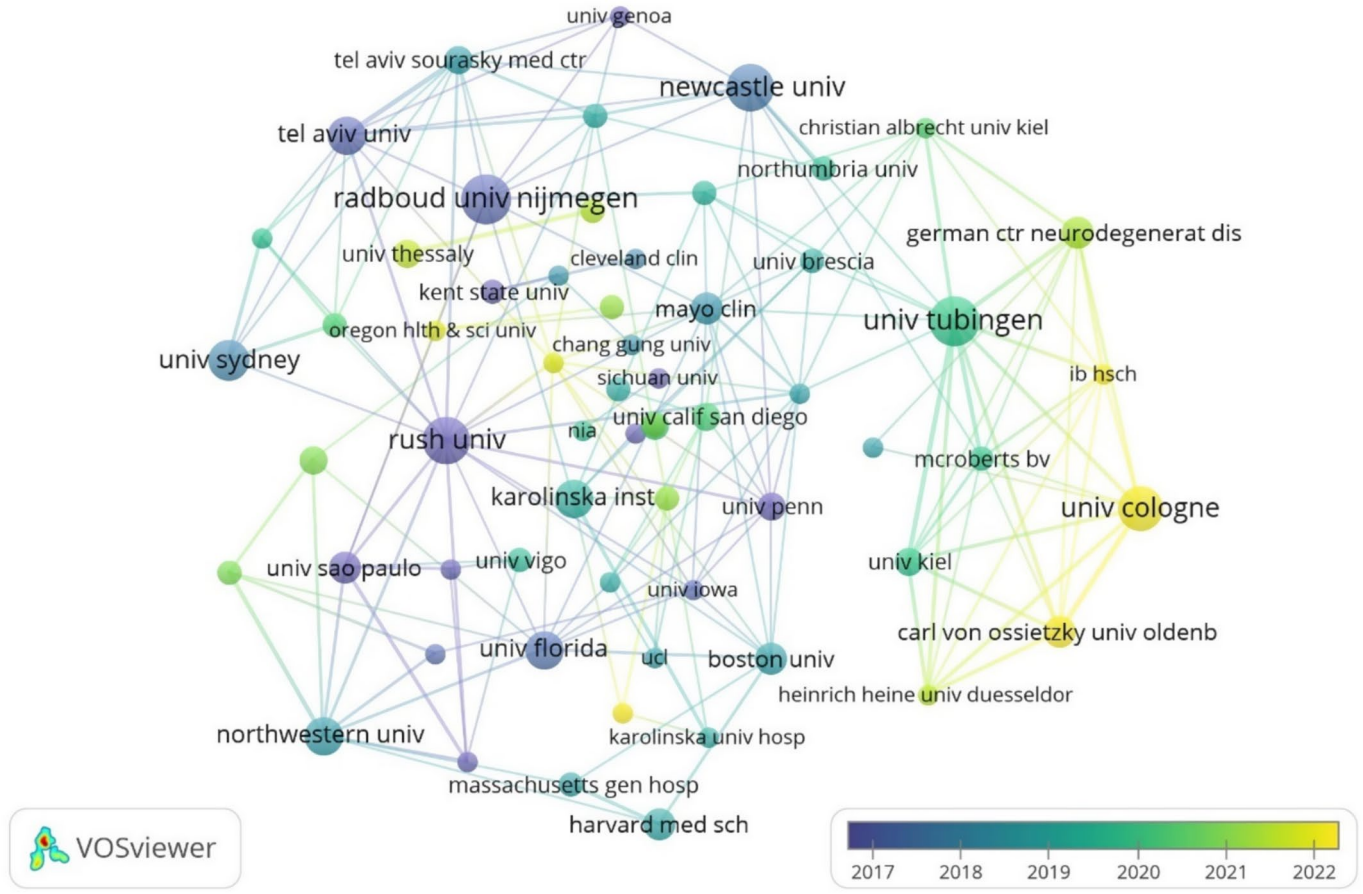
Time-based institutional collaboration map.

**Figure 7:**
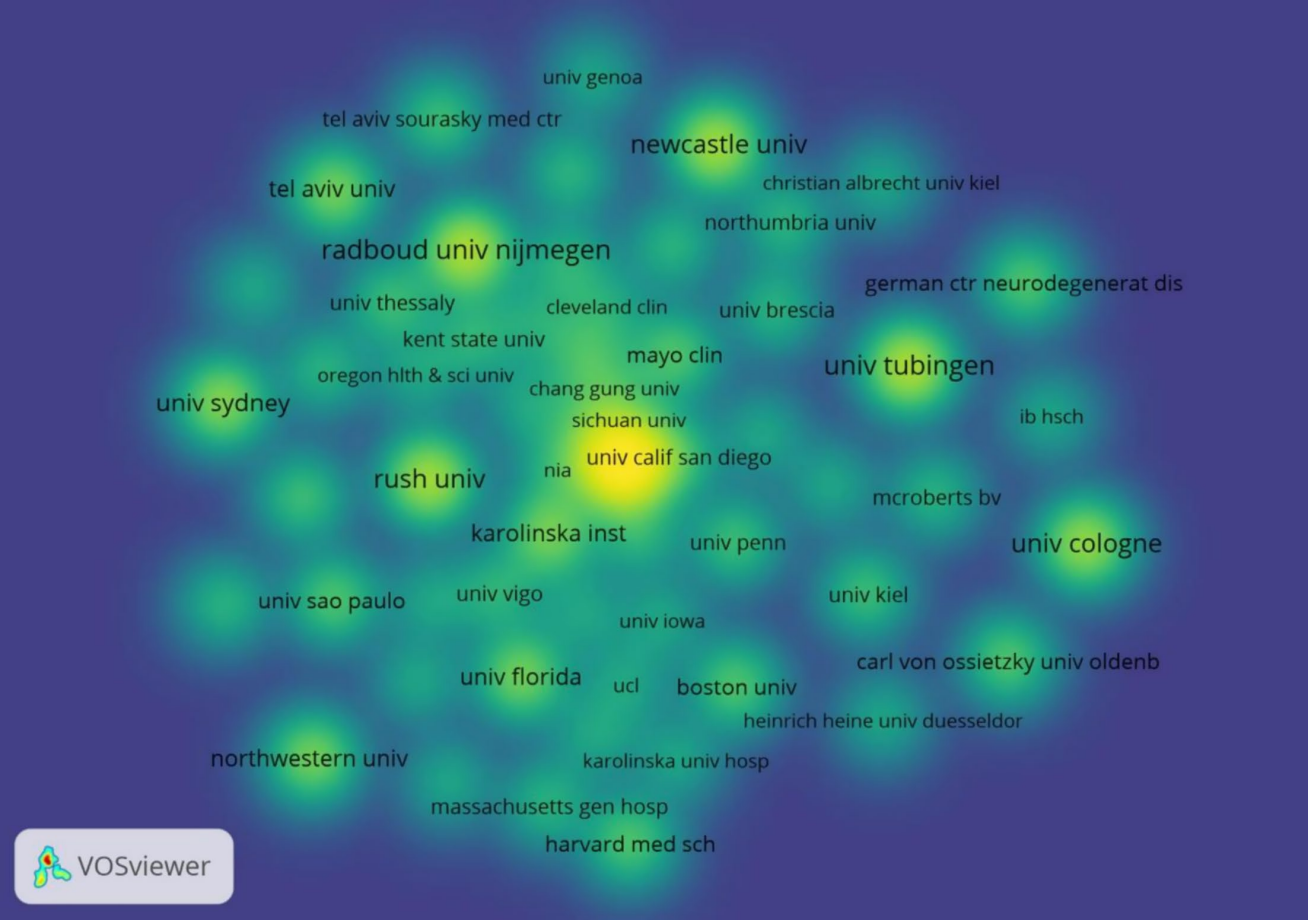
Institutional density map.

Among the issuing institutions, Eberhard Karls University of Tubingen was the most published and influential institution in the field with 15 papers. This was followed by Eberhard Karls University Hospital and University of California System with 13 and 12 papers, respectively. Newcastle University-UK, KU Leuven and Biogen were the centrally top three institutions with 0.25, 0.25, and 0.14, respectively, indicating that the institution has closer communication and collaboration with other institutions. The detailed information of the top 10 institutions in terms of the number of publications and centrality is shown in Table 2. In the institutional collaboration network, nodes with the same color indicate institutions that are more closely connected, as shown in Figure 5. The time-based institutional collaboration graph describes the temporal dynamics of institutional publications, as shown in Figure 6, Radboud University Nijmegen and Rush University initiated research in this area at an early stage. In the last 2 years University of Cologne has published a high number of papers in this field. In the institutional hotspot map, as shown in Figure 7, University of California San Diego University of London has a higher interest in this field.

**Table 2:** Top 10 institutions in terms of the number of publications.

Rangking	Publications	Institution	Centrality	Institution
1	15	Eberhard Karls University of Tubingen	0.25	Newcastle University - UK
2	13	Eberhard Karls University Hospital	0.25	KU Leuven
3	12	University of California System	0.14	Biogen
4	11	Radboud University Nijmegen	0.13	University of California System
5	10	Helmholtz Association	0.12	Northumbria University
6	10	US Department of Veterans Affairs	0.11	Boston University
7	10	German Center for Neurodegenerative Diseases (DZNE)	0.11	Case Western Reserve University
8	9	Harvard University	0.11	University of California San Diego
9	9	Rush University	0.1	University of London
10	9	Newcastle University - UK	0.1	Consejo Superior de Investigaciones Cientificas (CSIC)

### 3.4 Authors

**Figure 8:**
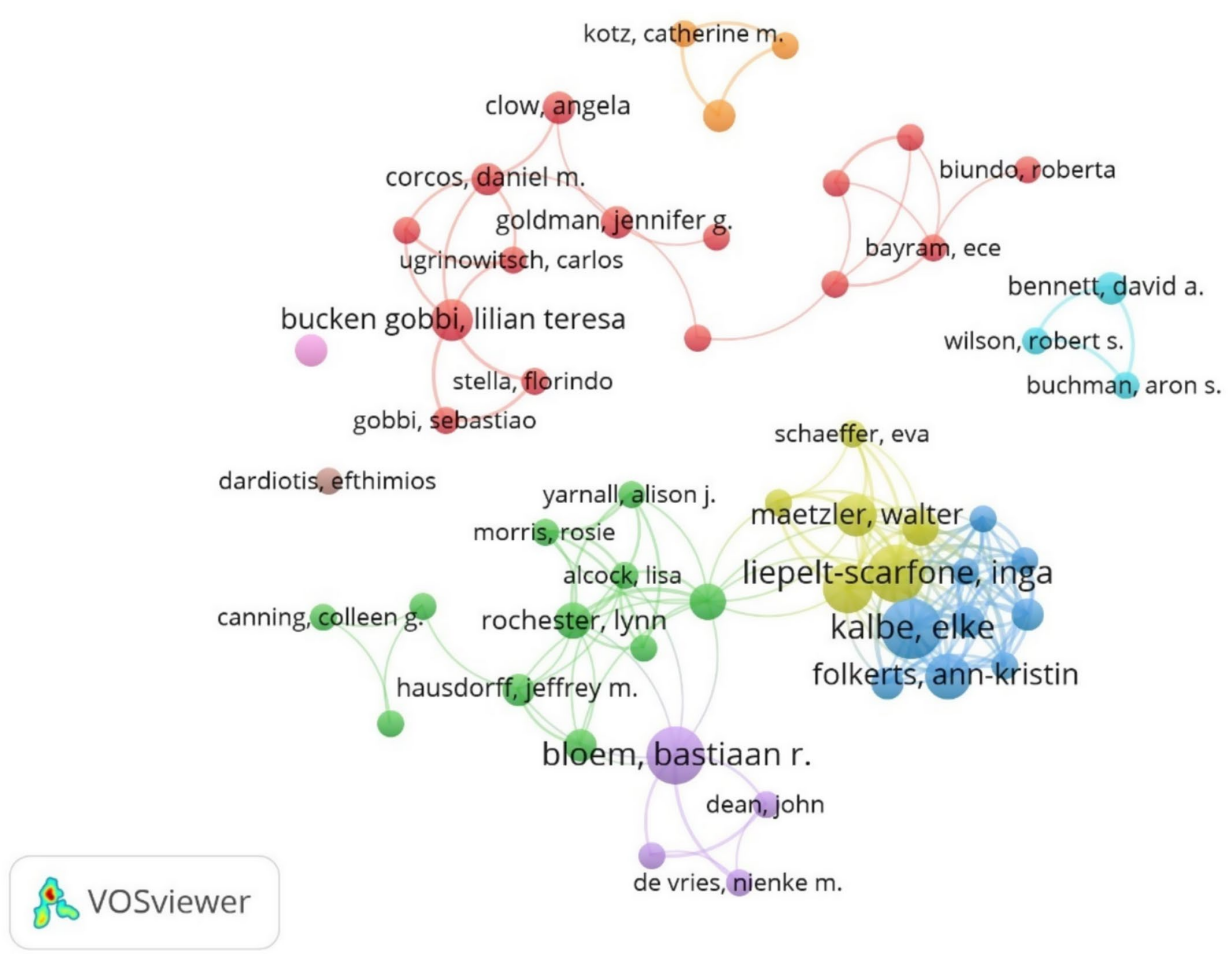
Cluster-based author collaboration map.

Among the issuing authors, the highest number of publications is Berg, Daniela (10), followed by Bloem, Bastiaan R (9). From the author network diagram, it can be seen that some authors form a close collaborative team and are more closely connected to each other, as shown in Figure 8. In terms of centrality, the author with high centrality is Berg, Daniela (0.04), and the top 10 authors in terms of publications and centrality are shown in Table 3.

**Table 3:** Top 10 authors in terms of the number of publications.

Rangking	Publications	Author	Centrality	Author
1	10	Berg, Daniela	0.04	Berg, Daniela
2	9	Bloem, Bastiaan R	0.03	Rochester, Lynn
3	7	Kalbe, Elke	0.03	Alcock, Lisa
4	5	Bucken gobbi, Lilian Teresa	0.03	Galna, Brook
5	4	Folkerts, Ann-Kristin	0.03	Schlenstedt, Christian
6	4	Bennett, David A	0.02	Bloem, Bastiaan R
7	4	Liepelt-scarfone, Inga	0.01	Kalbe, Elke
8	4	Rochester, Lynn	0.01	Liepelt-scarfone, Inga
9	3	Buchman, Aron S	0.01	Giladi, Nir
10	3	Giladi, Nir	0.01	Buttery, Sara Catherine

### 3.5 Keyword analysis

#### 3.5.1 Keyword co-occurrence analysis

**Figure 9:**
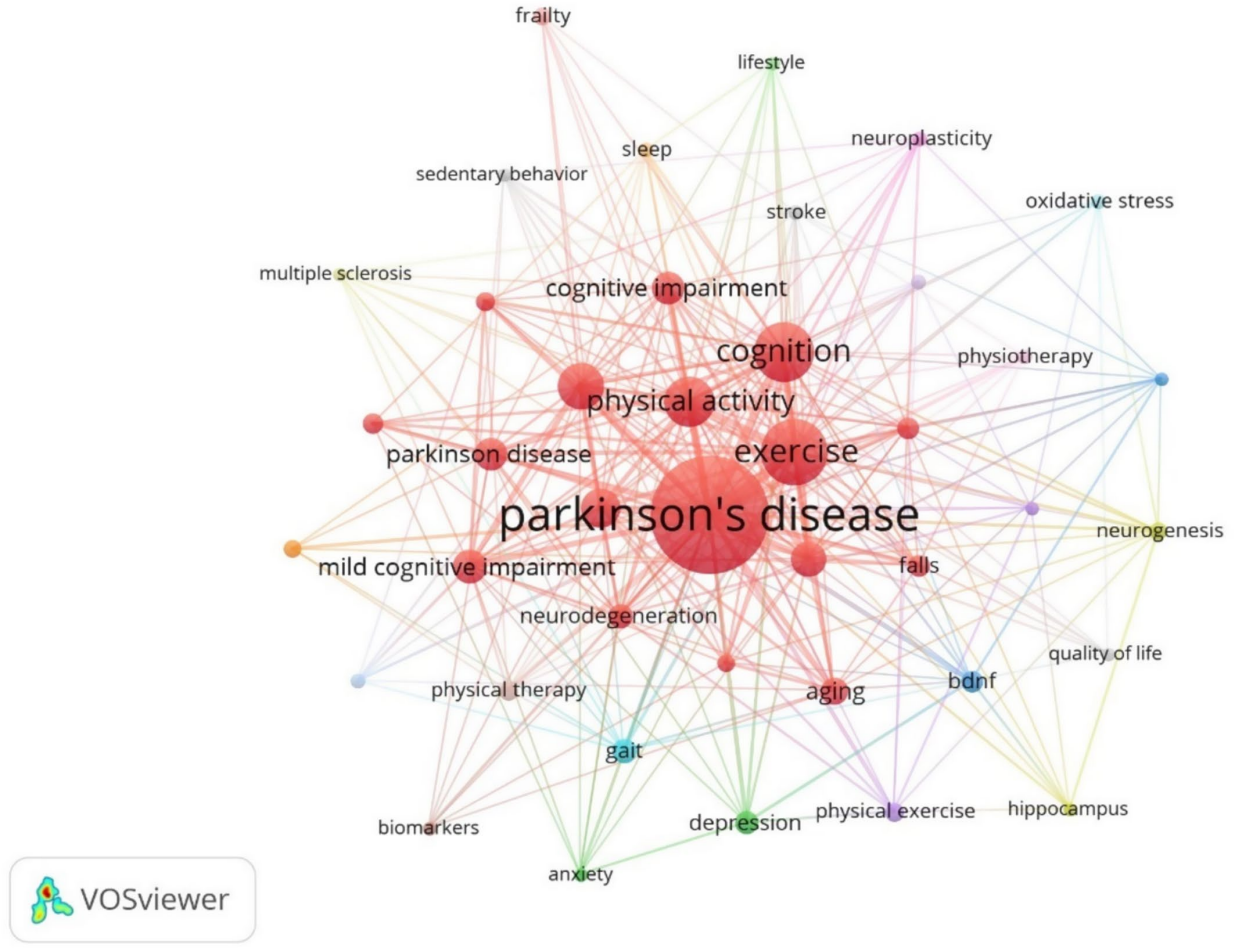
Cluster-based keywords collaboration map.

**Figure 10:**
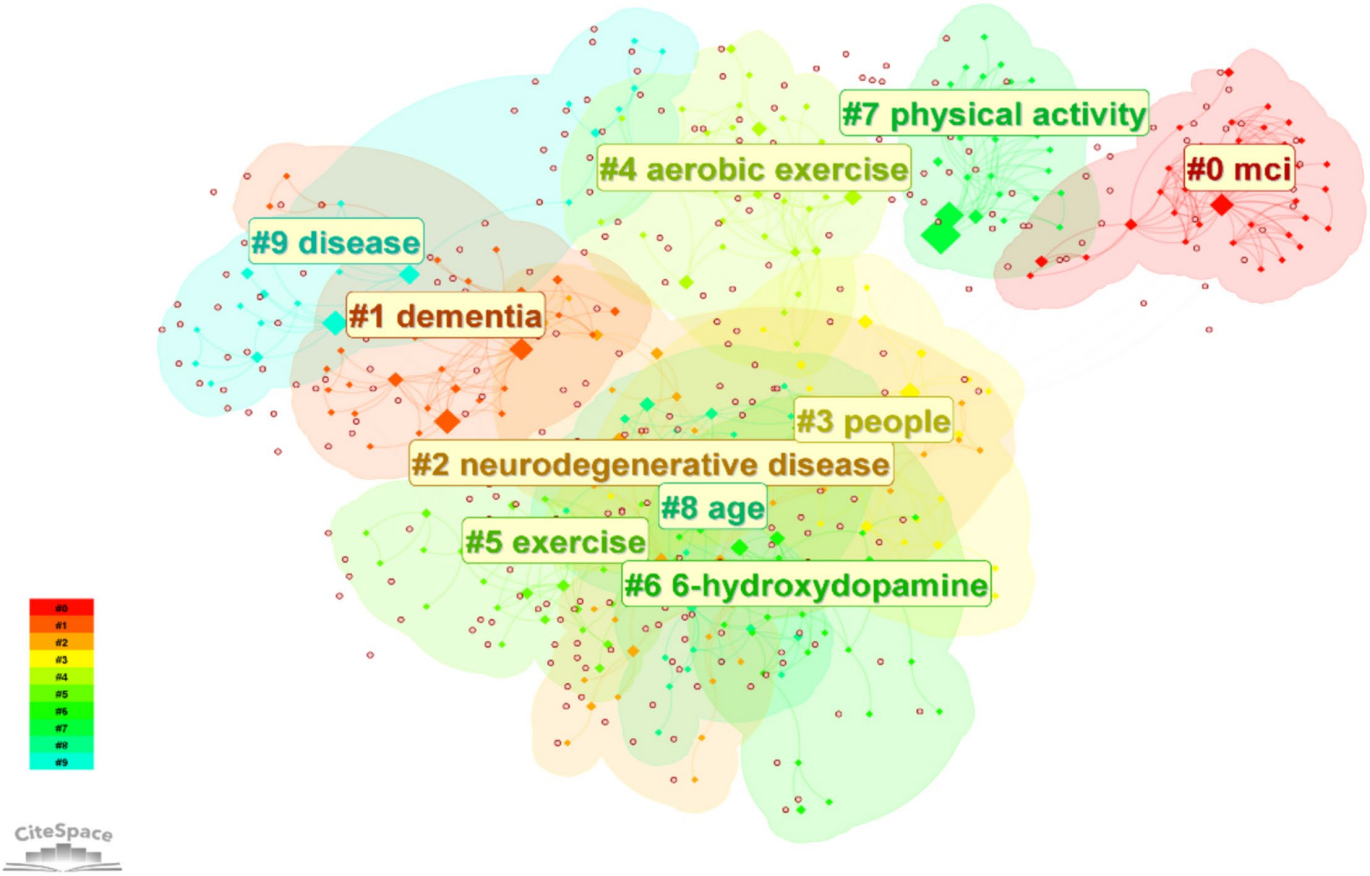
Keywords cluster map.

Keyword co-occurrence analysis can intuitively and comprehensively understand the research hotspots and trends in this field, as shown in Figure 10, the statistical results show that: from the point of view of the frequency of the keywords, PD appeared most frequently; followed by physical activity, cognitive impairment and Exercise etc. In terms of the centrality of keywords, the keyword with the highest centrality is cognitive decline, followed by PD, Dementia, Depression and aerobic exercise. The top 10 keywords in terms of frequency of occurrence and centrality are shown in Table 4 (Figure 9). From the keyword analysis, it can be seen that at present, the type of exercise that has been studied more in the field of Parkinson’ s cognitive impairment is aerobic exercise, and the importance of physical activity is getting more and more attention, and the main dysfunctions of cognitive impairment are cognitive decline and dementia, and the depressive symptoms are also getting more and more attention. According to the centrality of the keywords, the clinical efficacy of aerobic exercise to improve the cognitive function of Parkinson’ s patients as well as the mechanism of action research is the hot spot of research.

**Table 4:** Top 10 keywords of word frequency.

Ranking	Counts	Keywords	Centrality	Keywords
1	170	Parkinson’s disease	0.23	Cognitive decline
2	97	Physical activity	0.22	Parkinson’s disease
3	74	Cognitive impairment	0.2	Dementia
4	72	Exercise	0.2	Depression
5	64	Mild cognitive impairment	0.19	Aerobic exercise
6	59	Quality of life	0.17	Mouse model
7	55	Dementia	0.16	Basal ganglia
8	45	Older adults	0.15	Gait
9	44	People	0.13	Physical activity
10	33	Gait	0.12	Brain

Notably, some core mechanistic molecules closely related to the brain-gut microbiota axis, such as SCFAs, BDNF, and tumor necrosis factor-*α* (TNF-α), are not included in the top 10 high-frequency keywords due to their relatively low occurrence frequency in the included literature, which does not meet the software’s threshold for independent clustering. However, quantitative analysis of their co-occurrence with core themes shows that these mechanistic terms have obvious correlation with research hotspots: SCFAs co-occur 9 times with ‘brain-gut microbiota axis’(correlation coefficient *r* = 0.58), BDNF co-occurs 7 times with ‘cognitive impairment’ (*r* = 0.52), and TNF-α co-occurs 6 times with ‘neuroinflammation’ (*r* = 0.49). From the temporal trend, the annual co-occurrence frequency of the three terms with core themes has shown a continuous upward trend since 2018, indicating that mechanistic research focusing on these molecules is gradually becoming an emerging direction in the field, consistent with the research focus of exercise regulating PD cognitive function through the brain-gut microbiota axis.

#### 3.5.2 Keyword clustering analysis

Through the LLR algorithm in Citespace software to cluster analysis of high-frequency keywords, 10 representative keyword clustering labels were obtained, and the details of the clustered keywords are shown in Table 5. According to the results of the keyword clustering analysis, mild cognitive impairment and dementia are the dysfunctions of more concern. Aerobic exercise was the more studied intervention.

**Table 5:** Keywords cluster labels and main keywords.

Cluster number	Cluster label (LLR)	Value of contour	Year	Keywords
#0	Mild cognitive impairment	0.986	2011	Alzheimer’s disease; Parkinson; executive function;
#1	Dementia	0.9	2015	Treatment; cognitive impairment; mediation;
#2	Neurodegenerative disease	0.905	2018	Activities of daily living; aquatic exercise; functional laterality; macrophage;
#3	People	0.809	2014	Program; mild cognitive impairment; Community; chinese patients;
#4	Aerobic exercise	0.892	2013	Treadmill exercise; dance; motor; nonmotor symptoms;
#5	Exercise	0.808	2017	Mediterranean diet; mortality; s disease; Parkinson’;
#6	6-hydroxydopamine	0.867	2014	Accidental falls; brain; balance; oxidative stress;
#7	physical activity	0.936	2005	Parkinson’s disease; neurodegenerative diseases; glutamate; Parkinson’ disease;
#8	Age	0.907	2015	Memory; cognitive training; neuronal plasticity; visual processing;
#9	Disease	0.9	2016	Alzheimer’s disease; older adults; efficacy; qualitative

#### 3.5.3 Keyword time zone map

**Figure 11:**
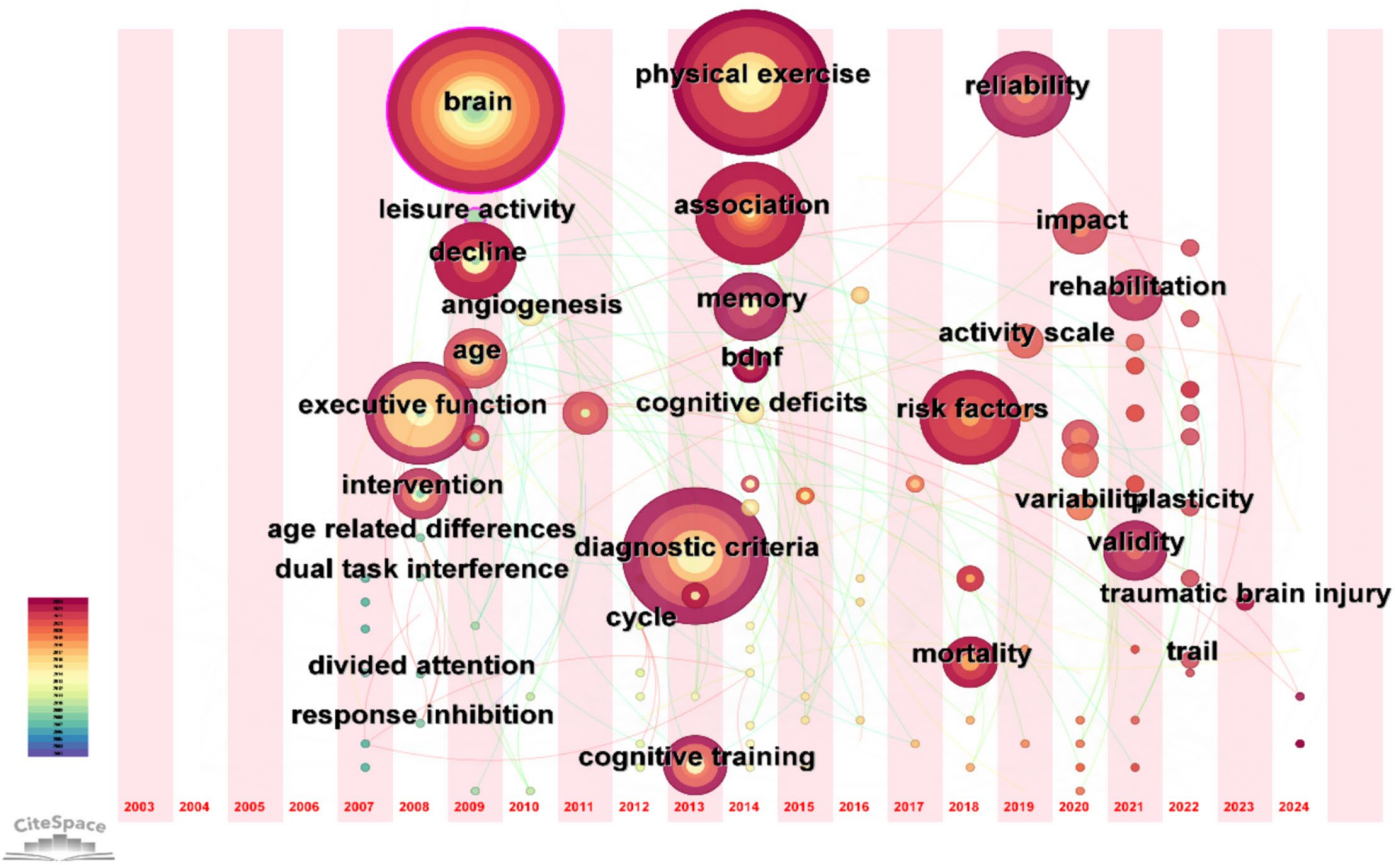
Timeline view of keywords.

The keyword time zone map can show the hotspots and trends in the research field through the historical evolution of keywords, as shown in Figure 11.

### 3.6 Highly cited analysis

**Figure 12:**
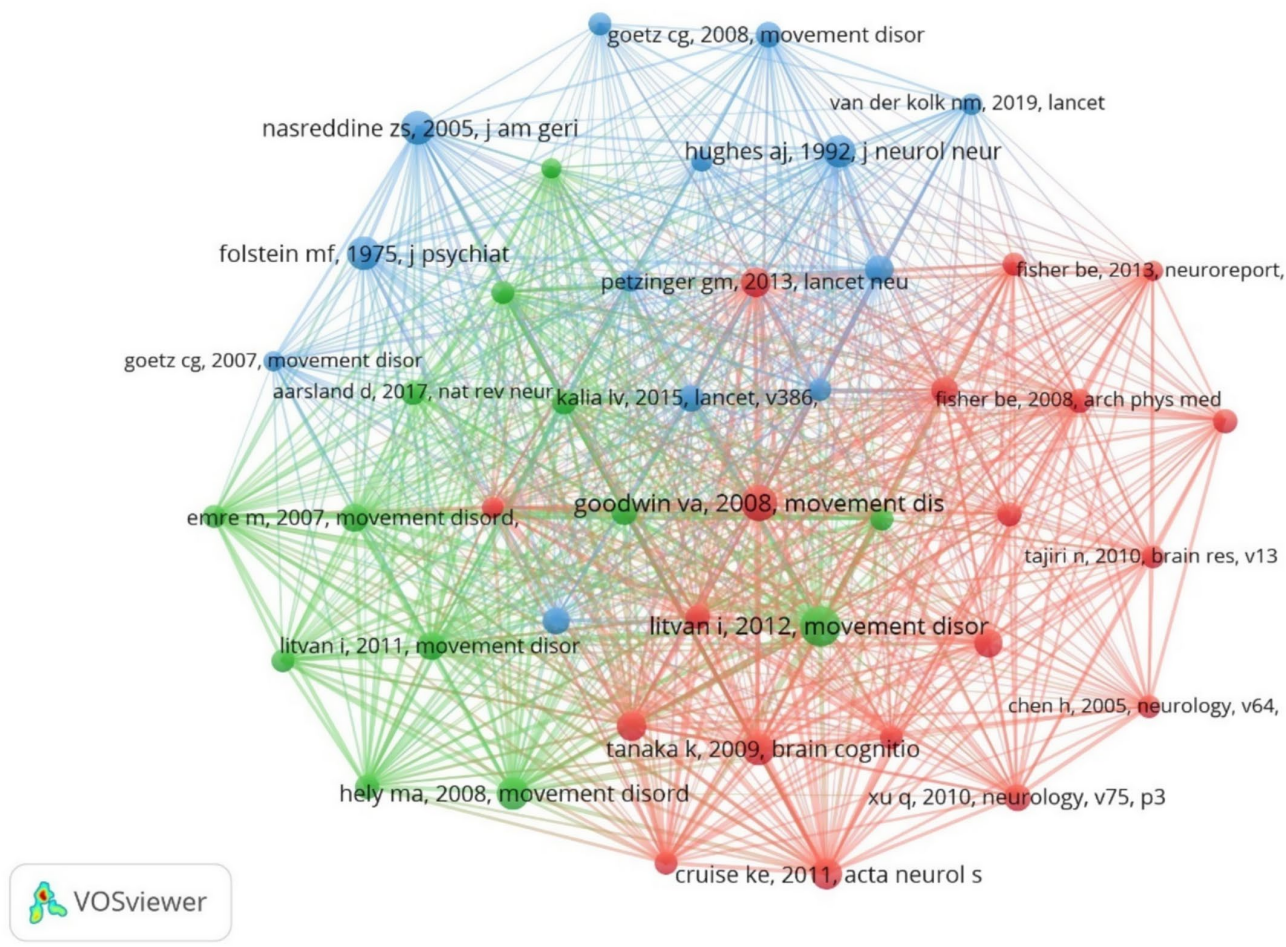
Cluster-based co-citation literature collaboration map.

Taking the cited literature as a node, the literature data were analyzed for literature high citation, as shown in Figure 12. The top 10 most frequently cited literatures are shown in Table 6. Highly cited literature often represents high impact literature in the field. Highly cited literature and high centrality literature mainly explore the latest research progress within the field. The article with the highest number of cited literature is “Exercise improves cognition in PD: the PRET-PD randomized, clinical trial” by David FJ (Chen and Song, 2019) published in the journal MOVEMENT DISORD, the clinical trial was a prospective, parallel-group, single-center trial. Fifty-one nondemented patients with mild to moderate Parkinson’ s were randomly assigned to either a modified fitness counting or progressive resistance exercise training group, and the results demonstrated that 24 months of modified fitness counting or progressive resistance exercise improved attention and working memory in nondemented patients with mild to moderate PD. Comprehensive analysis of the cited literature shows that the exercise interventions with higher attention are aerobic and high-intensity exercise, and the hotspots of the current research mainly focus on the clinical efficacy and mechanism of action of exercise to improve cognitive function in PD patients.

**Table 6:** The top 10 cited literature.

Ranking	Count	Author	Journal	Title
1	19	David et al., 2015	Movement Disord	Exercise improves cognition in Parkinson’ s disease: The PRET-PD randomized, clinical trial
2	15	Van der Kolk et al., 2019	Lancet Neurol	Effectiveness of home-based and remotely supervised aerobic exercise in Parkinson’ s disease: a double-blind, randomized controlled trial
3	14	Litvan et al., 2012	Movement Disord	Diagnostic criteria for mild cognitive impairment in Parkinson’ s disease: Movement Disorder Society Task Force guidelines
4	13	Cruise et al., 2011	Acta Neurol Scand	Exercise and Parkinson’ s: benefits for cognition and quality of life
5	13	Aarsland et al., 2021	Nat Rev Dis Primers	Parkinson disease-associated cognitive impairment
6	12	Hindle et al., 2013	Movement Disord	Nonpharmacological enhancement of cognitive function in Parkinson’ s disease: a systematic review
7	11	Schenkman et al., 2018	JAMA Neurol	Effect of High-Intensity Treadmill Exercise on Motor Symptoms in Patients With *De Novo* Parkinson Disease: A Phase 2 Randomized Clinical Trial
8	11	Aarsland et al., 2017	Nat Rev Neurol	Cognitive decline in Parkinson disease
9	10	Ahlskog, 2011	Neurology	Does vigorous exercise have a neuroprotective effect in Parkinson disease?
10	10	Kalia and Lang, 2015	Lancet	Parkinson’ s disease

### 3.7 Journal co-citation

**Figure 13:**
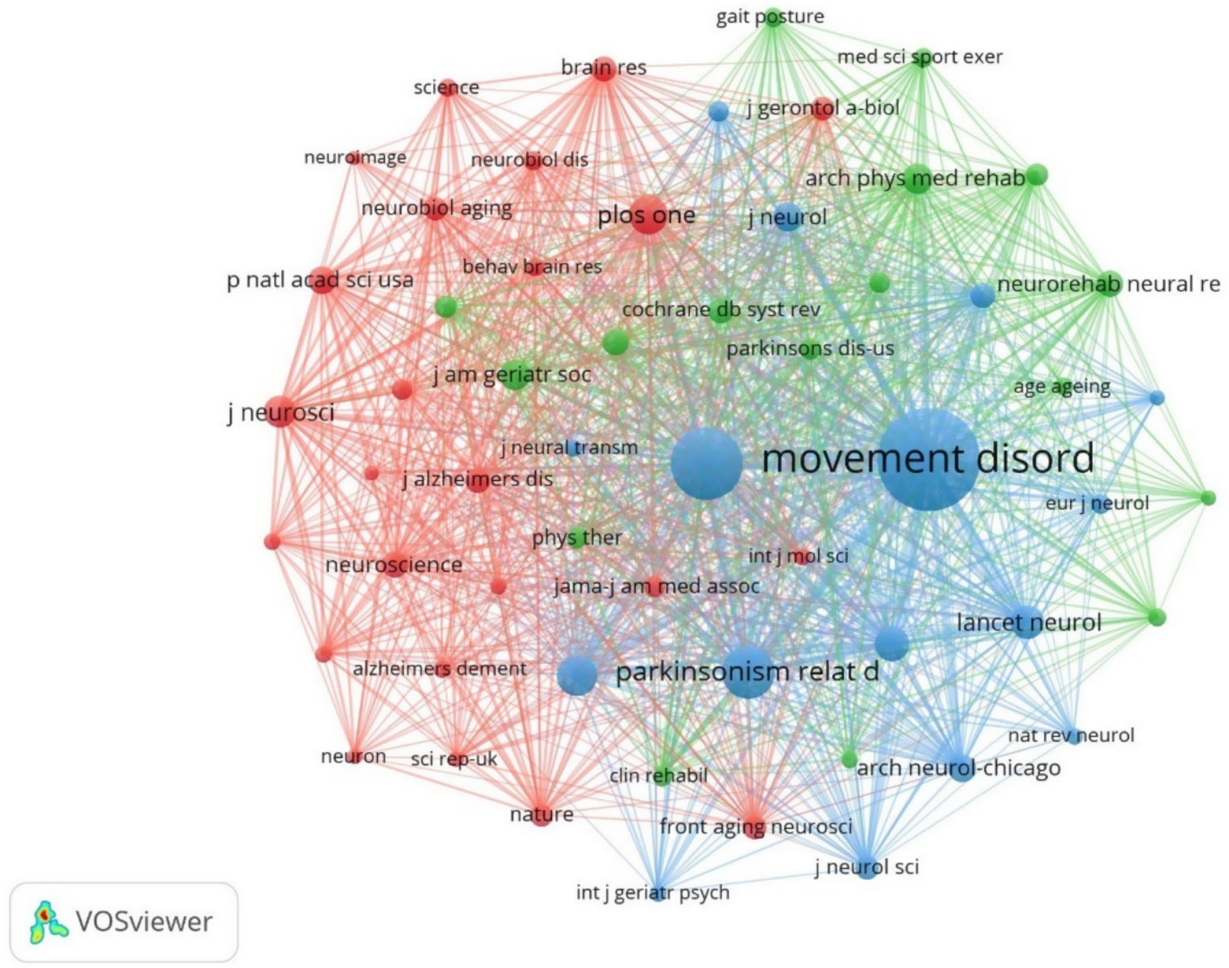
Cluster-based Journal co-citation collaboration map.

Journal co-citation analysis of literature data from Web of science core collection database using cited journals as nodes is shown in Figure 13. The top 5 journals in terms of citation frequency are shown in Table 7. The journal with the highest citation frequency is NEUROLOGY (259). The journal with the highest centrality was ADV NEUROL (0.83). Most of the combined cited journals were medical and sports.

**Table 7:** Top 10 cited journals.

Ranking	Counts	Journal	Centrality	Journal
1	259	Neurology	0.83	Adv Neurol
2	257	Movement Disord	0.25	Acta Physiol Scand
3	202	Parkinsonism Relat D	0.15	Acta Neuropathol
4	173	PLoS One	0.12	Arch Neurol-Chicago
5	172	J Neurol Neurosur PS	0.11	Age Ageing
6	169	Lancet Neurol	0.1	Physiol Behav
7	143	Brain	0.09	Brit J Psychiat
8	137	J Neurol	0.09	Arch Gen Psychiat
9	126	J Am Geriatr Soc	0.08	Behav Brain Res
10	110	J Parkinson Dis	0.07	Acta Neurol Scand

### 3.8 Journal overlay

**Figure 14:**
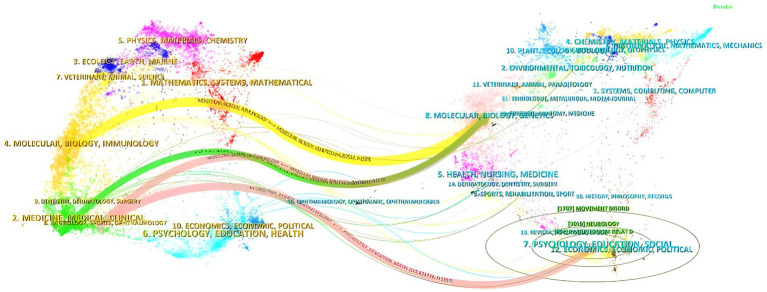
The dual-map overlay of journals.

The journal bimap overlay shows the position of a research topic relative to the major research disciplines, visualizing the research dynamics of the disciplines through the flow of information at the journal level, see Figure 14. The journal bimap overlay consists of two main components: the citing journals on the left and the cited journals on the right. There are four main citation paths in the journal bimap overlay. The yellow path indicates: journals in the field of molecular/biology/immunology are usually cited by molecular/biology/genetics (*z* = 4.257511, *f* = 1,529). The green path indicates that: journals in the field of medicine are influenced by journals in the field of molecular/biology/genetics (*z* = 1.8532717, *f* = 758). The red path indicates that: journals in the field of Neurology/Kinesiology/Ophthalmology are influenced by journals in the field of Molecular/Biology/Genetics (*z* = 2.9696327, *f* = 1,116) and Psychology/Education/Society (*z* = 2.629738, *f* = 1,007).

Based on the bibliometric analysis of relevant publications from 1999 to 2024, we systematically summarized the global research status, hotspots, and development trends of exercise intervention for cognitive impairment in Parkinson’s disease (PD). The results indicate that the number of publications in this field has shown a continuous growth trend, peaking in 2022, with the United States leading in research output, and Eberhard Karls University of Tubingen and author Berg, Daniela being the most influential institution and researcher, respectively. Keyword co-occurrence and clustering analyses revealed that mild cognitive impairment, dementia, and depression are the core cognitive-related symptoms of concern, while aerobic exercise is the most extensively studied exercise modality. Additionally, the brain-gut microbiota axis has emerged as a key research focus in exploring the mechanism of exercise-induced cognitive improvement. These bibliometric findings not only reflect the current research landscape and academic focus in this field but also provide a solid foundation for further in-depth discussion of the underlying molecular mechanisms, clinical implications, and future research directions. The following discussion will integrate these core results with existing clinical and basic research evidence to elaborate on the potential pathways by which exercise modulates cognitive function in PD through the brain-gut microbiota axis, analyze the differential effects of various exercise modalities, and explore the clinical application value of personalized exercise interventions. For “SCFAs,” “BDNF,” and “TNF-*α*,” although they are core mechanistic molecules in the field, their absolute occurrence frequency in the 332 included publications does not meet the threshold for independent clustering in VOSviewer and CiteSpace software the software’s default clustering standard requires a minimum occurrence frequency of 15 times for keywords. While bibliometric analysis effectively identifies the dominant research landscape, it may fail to capture emerging hotspots and cuttingedge directions that have not yet formed sufficient clustering patterns in the literature. Consequently, this approach may overlook potential future research trends and the latest mechanistic discussions that are still in the early developmental stage.

## 4 Discussion

### 4.1 Current status of research

The increasing recognition of exercise in PD research in recent years (as reflected by the rising annual publication output, peaking in 2022) is closely associated with the limitations of existing pharmacological approaches for non-motor symptoms such as cognitive impairment, depression, and sleep disturbances. Currently, central acetylcholinesterase inhibitors and NMDA receptor antagonists can only temporarily relieve dementia symptoms without modifying the disease course (Annesi, 2019), and there is a lack of targeted drugs for PD-related depression and cognitive decline. In contrast, exercise, as a safe, low-cost, and widely accessible non-pharmacological intervention, not only improves motor function but also exerts multi-dimensional benefits on non-motor symptoms. Moreover, pharmacological treatments often carry risks of adverse reactions (e.g., dyskinesia induced by long-term L-DOPA use), while exercise has minimal side effects and can be personalized according to patients’ physical conditions. These advantages have driven the surge in research interest in exercise-based interventions for PD, making it a promising complement or alternative to pharmacological therapies for non-motor symptom management.

From the annual number of publications and growth trend from 2003 to 2024 in general, the number of publications shows an increasing trend, and in recent years, the growth rate is faster, the number of articles is higher, indicating that the role of exercise and sports exercise in improving the cognition of Parkinson’ s patients is widely recognized and concerned, and the research in this field reaches the peak in the number of publications in 2022. The country with the highest number of publications is the United States, with a total of 117 publications, followed by the United Kingdom and Germany, with 38 and 34 publications, respectively. In terms of the centrality of the issuing countries, Germany, the UK, and Italy have high centrality of 0.47, 0.3, and 0.27, respectively, indicating that the above countries maintain close cooperation with other countries/regions. From the heat map of issuance, the research heat of the United States in this field is higher, and the United States has advanced instrumentation and specialized talents engaging in this area of research, and has achieved certain research results in this field. Most of the countries with more publications and higher centrality are developed countries, which have accumulated rich experience in sports medicine and Parkinson’ s research and contributed to the research and development of this field, so as to better explain the efficacy and mechanism of the effect of exercise on the cognitive function of Parkinson’ s patients.

Among the issuing institutions, Eberhard Karls University of Tubingen is the most prolific and influential institution in the field with 15 papers. Radboud University Nijmegen and Rush University initiated research in this field at an early stage. In the last 2 years University of Cologne has published a high number of papers within the field. The institutional hotspot map shows that University of California San Diego University of London has a higher interest in this field. Among the issuing authors, the highest number of publications is Berg, Daniela (10) followed by Bloem, Bastiaan R (9). The author network diagram shows that some of the authors are more closely connected by forming close collaborative teams with each other. In terms of centrality, the author with higher centrality is Berg, Daniela (0.04). The authors with more publications are mainly concentrated in developed countries, and the authors communicate and cooperate with each other to promote the development of research in this field. The journal with the highest citation frequency is NEUROLOGY (259 articles). It is the official journal of the American Academy of Neurology, a leading peer-reviewed journal focusing on clinical neurology research. The main goal is to advance the field of neurology by publishing new basic and clinical research. The journal with the highest centrality is ADV NEUROL (0.83). It is a peer-reviewed, open access academic journal published by AccScience Publishing, Singapore, which is dedicated to the publication and dissemination of basic, clinical, and translational medicine research related to neurological disorders, with the aim of advancing the understanding of neurological-related disorders and providing a high-quality platform for scholarly communication in the field of neurology. Most of the comprehensively cited journals are in the medical and sports categories.

### 4.2 Research hot spots

Parkinson’ s disease-related mild cognitive impairment (PD-MCI) constitutes a prodromal stage of PD dementia. It involves progressive dysfunction across multiple brain regions, which exhibit selective vulnerability to neurodegeneration and demonstrate differential responses to exercise-mediated signaling pathways. Early loss of cholinergic neurons in the Nucleus Basalis of Meynert (NBM) disrupts acetylcholine homeostasis. Exercise-induced vascular endothelial growth factor (VEGF) and estrogen exert neuroprotective effects via trophic support and the ERβ signaling pathway. Degeneration of noradrenergic neurons in the Locus Coeruleus (LC), often preceding nigral pathology, contributes to cognitive and attentional deficits. Exercise-derived brain-derived neurotrophic factor (BDNF) promotes neuronal survival in the LC, while appropriate cortisol fluctuations fine-tune neurotransmitter release. Structural and functional abnormalities within the prefrontal cortex (PFC) and hippocampus impair executive function and memory. Exercise-induced BDNF, insulin-like growth factor-1 (IGF-1), and SCFAs enhance synaptic plasticity, neurogenesis, and neurotransmission through distinct receptor pathways. Dysfunction of the basal ganglia–thalamocortical circuit reduces information processing speed. Exercise-associated dopamine and SCFAs can enhance signal transmission and suppress neuroinflammation, respectively, thereby aiding in the restoration of circuit function.

According to keyword co-occurrence analysis and keyword cluster analysis, it can be concluded that the symptoms that attract more attention in current research on cognitive impairment in PD patients are mild cognitive impairment, dementia, and depression. Among the many studies related to exercise methods, aerobic exercise has always been a hot research topic in this field. In terms of mechanism research, exercise’s regulation of Parkinson’ s cognitive ability through the brain-gut axis has been a hot topic in recent years. Combined with the quantitative analysis of mechanistic keywords, although SCFAs, BDNF, and TNF-*α* do not meet the threshold for independent clustering, their increasing co-occurrence frequency with core themes (such as brain-gut microbiota axis, cognitive impairment, neuroinflammation) further confirms that the regulation of inflammatory response (mediated by TNF-α), neurotrophic factor expression (mediated by BDNF), and metabolite metabolism (mediated by SCFAs) is the core mechanism of exercise improving PD cognitive impairment. This is consistent with the results of clinical and basic research summarized later, reflecting the organic combination of bibliometric analysis and mechanistic exploration in this study.

#### 4.2.1 Differential effects of various exercise modalities on gut microbiota and intestinal permeability

Aerobic exercise, characterized by moderate intensity and sustained duration (e.g., jogging, swimming), serves as a fundamental approach to regulating intestinal health. Studies have confirmed that regular aerobic exercise significantly enhances the alpha diversity of the gut microbiota, increases the abundance of beneficial bacteria such as Bifidobacterium and Akkermansia, and optimizes the balance between the Firmicutes and Bacteroidetes phyla (Shan et al., 2025). Its beneficial effect on intestinal permeability is well-established: by upregulating the expression of tight junction proteins such as ZO-1 and Claudin-1, aerobic exercise strengthens intestinal barrier integrity, reduces serum levels of LPS and diamine oxidase (DAO), and lowers the risk of bacterial translocation (Thangaleela et al., 2022).

Resistance exercise (e.g., weight training) exerts a more focused impact on the modulation of functionally specific microbial populations. Although this type of exercise has a moderate effect on overall microbial diversity, it significantly increases the abundance of butyrate-producing bacteria such as *Faecalibacterium prausnitzii* (Caballero-Villarraso et al., 2025). Butyrate, as the primary energy source for colonocytes, directly suppresses intestinal inflammation and promotes barrier repair (Pang et al., 2026). Regarding intestinal permeability, resistance exercise indirectly improves barrier function by modulating mucin synthesis and inflammatory cytokine levels, demonstrating particular value in maintaining intestinal health in aging populations (Suda and Matsuda, 2022).

The effects of endurance exercise (e.g., long-distance running, cycling) exhibit intensity dependency. Moderate-intensity long-term endurance training consistently enriches beneficial bacteria including Prevotella and *Faecalibacterium prausnitzii*, boosts short-chain fatty acid (SCFA) production, and enhances intestinal barrier function (Noble et al., 2025). However, extreme endurance exercise (e.g., ultramarathon) may trigger stress responses, leading to transient gut microbiota dysbiosis accompanied by a temporary increase in intestinal permeability, necessitating proper recovery to prevent long-term damage (Zheng et al., 2024).

The influence of high-intensity interval training (HIIT) is bidirectional. Short-term HIIT can rapidly increase the abundance of beneficial bacteria such as Lactobacillus (Zheng et al., 2024), while long-term adherence promotes the restoration of microbial diversity and optimizes metabolic pathways (Guo et al., 2023). However, due to its high-intensity nature, HIIT may cause transient changes in intestinal blood flow, leading to a temporary rise in intestinal permeability. Overtraining may further induce microbiota imbalance and inflammatory responses, underscoring the need for strict control over exercise frequency and intensity ratios (Xue et al., 2020).

In summary, different types of exercise differentially influence the gut microbiota and intestinal permeability by modulating intestinal motility, metabolic byproducts, and immune status (Caballero-Villarraso et al., 2025) Moderate-intensity aerobic exercise and regular resistance exercise represent optimal strategies balancing safety and efficacy, whereas endurance exercise and HIIT require scientifically tailored implementation based on individual tolerance to achieve precise regulation of intestinal health (Shan et al., 2025).

#### 4.2.2 Molecular mechanisms by which the brain-gut flora axis affects cognition

##### 4.2.2.1 The impact of dysbiosis across multiple mouse models of PD

Dysbiosis exerts consistent pathological effects across various PD mouse models, with distinct but overlapping mechanisms linked to cognitive impairment. In rotenone-induced PD models, intestinal flora imbalance disrupts the intestinal barrier and blood–brain barrier (BBB), enabling bacterial endotoxin (lipopolysaccharide, LPS) to infiltrate brain tissue, activate the TLR4/NF-κB signaling pathway, trigger neuroinflammation, and ultimately promote cognitive dysfunction (Guo et al., 2023). Different PD models, such as 6-hydroxydopamine-induced models and *α*-synuclein overexpression models, exhibit variations in neuroinflammation levels and microbiome composition, which should be considered when interpreting the regulatory effects of exercise (Xue et al., 2020). Additionally, the sleep-deprivation model (chronic partial sleep deprivation: 8 h/day for 4 weeks) induces cognitive deficits by disrupting neuroendocrine homeostasis and intestinal flora balance (Chen Y. et al., 2025). Notably, sleep deprivation impairs glymphatic system function-an essential pathway for clearing brain metabolic waste-leading to accumulation of α-synuclein aggregates and enhanced neuroinflammation. In this model, intestinal flora disturbance further exacerbates the upregulation of inflammatory factors and neuronal apoptosis, while supplementing 5-hydroxytryptamine (5-HT) or regulating intestinal flora can reverse these pathological changes and cognitive defects (Pang et al., 2026). Collectively, these mouse models confirm that gut microbiota dysbiosis is a conserved driver of neuroinflammation and cognitive impairment in PD-related pathogenesis.

##### 4.2.2.2 The effects of gut microbiota and its components on the central nervous system

Intestinal flora and its metabolites are key regulators of central nervous system (CNS) function, with profound impacts on neurological health and cognition (Yassin et al., 2025). Short-chain fatty acids (SCFAs), the primary metabolites of intestinal probiotic fermentation of dietary fiber, can cross the BBB to modulate the inflammatory state, neuroplasticity, and neuronal metabolism of the CNS (Trisal et al., 2025). For example, icariin significantly improves D-galactose-induced cognitive impairment by regulating intestinal flora and promoting SCFA production (Li S. et al., 2025). SCFAs also reduce neuroinflammation and cognitive impairment in Alzheimer’s disease model mice via the cAMP-PKA-CREB-HDAC3 signaling pathway (Zhang et al., 2022). In rodent models of PD (specifically the rotenone-induced model), SCFA deficiency and impaired intestinal barrier function are closely associated with cognitive decline, as they promote the release of inflammatory factors and activation of pro-inflammatory microglia in the substantia nigra and cortex-brain regions critical for dopaminergic function and cognitive regulation (Liu et al., 2021). Given the dual (pro-inflammatory and neuroprotective) phenotypes of microglia, the activation of pro-inflammatory microglia in these regions further damages neurons and exacerbates cognitive impairment (Guo et al., 2023). Additionally, gut flora modulates the activation status of microglia, thereby regulating neuroinflammation and synaptic plasticity, which have profound impacts on cognitive function (He et al., 2023).

##### 4.2.2.3 The consequences of dysbiosis on gut health, including gastrointestinal symptoms

Gut microbiota dysbiosis directly impairs gut health, with gastrointestinal symptoms serving as potential early predictors of cognitive decline in PD (Zhang et al., 2025). Gastrointestinal symptoms such as constipation-common non-motor symptoms of PD-are closely linked to intestinal flora imbalance (Zhang et al., 2025). This association is mediated through the gut-brain axis: dysbiosis exacerbates intestinal inflammation and promotes *α*-synuclein aggregation in the gut, which then retrogradely spreads to the CNS via the vagus nerve, causing damage to cognition-related brain regions (Zhang et al., 2025). Moreover, dysbiosis disrupts intestinal barrier integrity, leading to increased permeability and translocation of bacterial products (e.g., LPS) into the circulation, which further amplifies systemic inflammation and contributes to gut-brain axis dysfunction (Li C. et al., 2025). These gut-related pathological changes not only worsen gastrointestinal symptoms but also create a pro-inflammatory microenvironment that accelerates neuroinflammation and cognitive decline in PD.

##### 4.2.2.4 Human and clinical studies in PD patients

Clinical studies confirm that gut microbiota dysbiosis is closely associated with cognitive impairment in PD patients (Wang et al., 2024). PD patients with cognitive impairment exhibit significantly reduced intestinal flora alpha diversity, with decreased abundances of beneficial bacterial genera such as *Faecalibacterium prausnitzii*, Clostridia, and Ruminococcaceae-abundances that are positively correlated with cognitive function scores (Wang et al., 2024). Notably, PD-related cognitive impairment is consistently linked to depleted levels of SCFA-producing bacteria (e.g., Bacteroides and Prevotella) (Xue et al., 2020). This reduction in SCFA-producing taxa may diminish the synthesis of anti-inflammatory metabolites, exacerbate intestinal inflammation, disrupt gut barrier integrity, and thereby accelerate neuroinflammation and cognitive decline (Xue et al., 2020). In contrast, accumulating evidence indicates increased abundances of Lactobacillus and *Akkermansia muciniphila* in PD patients (He et al., 2023), with preclinical and clinical data suggesting these taxa may exert partial neuroprotective effects by regulating gut epithelial barrier function and modulating systemic inflammatory responses-though their direct correlation with cognitive function outcomes remains to be fully elucidated (He et al., 2023). These clinical findings align with experimental model results, confirming the critical role of gut microbiota dysbiosis in PD-related cognitive impairment.

#### 4.2.3 The mechanism of action between exercise and the brain-gut microbiota axis

As an important lifestyle intervention, exercise can significantly affect the composition of the intestinal microbiota and its metabolites, and then have a profound impact on the nervous system through the brain-gut microbiota axis. SCFAs produced by intestinal microbiota metabolism are one of the key mediators for exercise to improve neurological function. SCFAs mainly include acetic acid, propionic acid and butyric acid, which can penetrate the blood–brain barrier, regulate neuroinflammatory response and neuronal metabolism, and exert neuroprotective effects (He et al., 2023). The mechanism by which exercise promotes the production of intestinal SCFAs mainly involves the exercise-induced increase in the diversity of intestinal flora and the increase in the abundance of beneficial flora, such as Akkermansia, Lachnospiraceae, etc. These flora can enhance the synthesis ability of SCFAs (Noble et al., 2025).

SCFAs regulate immune cell function and reduce inflammation levels in the CNS by activating G protein-coupled receptors (such as GPR41, GPR43) and inhibiting histone deacetylase (HDAC) activity, thereby improving cognitive function and neuronal survival. In addition, SCFAs can also enhance mitochondrial function, promote neuronal energy metabolism, and support neural plasticity and synaptic function (Trisal et al., 2025). Exercise-induced changes in intestinal metabolism are not limited to SCFAs, but also include bile acids, tryptophan metabolites, etc. (Vazquez-Medina et al., 2024). These metabolites are involved in the synthesis and regulation of neurotransmitters and further promote nervous system health (Xiao et al., 2025).

Exercise-induced metabolomics research shows that long-term exercise can regulate key neurometabolic pathways in serum and brain, such as the levels of neurotransmitters such as glutamate and *γ*-aminobutyric acid (GABA), and improve cognitive ability and learning and memory functions (Shan et al., 2025). Through multi-omics analysis, it was found that the diversity of intestinal flora of mice in the exercise group was significantly enhanced, and the levels of related metabolites were significantly changed, showing metabolic characteristics that are beneficial to neuroprotection (Shan et al., 2025). In addition, exercise promotes muscle secretion of myokines, such as Irisin, which can reduce neuroinflammation and delay cognitive decline by regulating intestinal flora and its metabolites (Zheng et al., 2024).

It is worth noting that exercise mode and intensity have different effects on intestinal flora and metabolites. Moderate-intensity continuous exercise (MICT) is generally beneficial to promoting intestinal barrier function and the production of beneficial metabolites, while high-intensity interval training (HIIT) may increase intestinal permeability and endotoxin (such as LPS) levels, bringing the risk of cognitive function decline (Su and Su, 2025). Therefore, scientific and reasonable exercise prescription is particularly important to optimize intestinal metabolites and their effects on the nervous system.

#### 4.2.4 Mechanism of improving cognitive ability based on the brain-gut axis: inflammatory factors and immune regulatory pathways

In the pathological mechanism of cognitive impairment in PD, the elevated expression of inflammatory factors such as tumor necrosis factor-*α*(TNF-α) and interleukin-6 (IL-6) represents a central event, which is closely linked to immune dysregulation mediated by the gut microbiota, collectively driving a “gut dysbiosis-immune activation-neuroinflammation-cognitive decline” cascade (Li C. et al., 2025).

The immune system, particularly regulatory T cells (Tregs), plays a crucial role in maintaining gut immune homeostasis (Chen Y. et al., 2025). The gut microbiota is an essential regulator of immune balance: it promotes the differentiation and maturation of anti-inflammatory Tregs, which suppress excessive pro-inflammatory responses and maintain intestinal immune quiescence (Chen Y. et al., 2025). Beneficial bacteria, such as short-chain fatty acid (SCFA)-producing species, are key mediators of this process-their metabolites enhance Treg proliferation and function, thereby limiting the release of pro-inflammatory cytokines and preserving gut barrier integrity (Suda and Matsuda, 2022). Additionally, the gut microbiota modulates innate immune responses by regulating the activation of intestinal epithelial cells and immune cells (e.g., macrophages, dendritic cells), further reinforcing immune homeostasis at the gut-brain interface (Caballero-Villarraso et al., 2025).

In PD patients, gut microbiota dysbiosis disrupts this immune regulatory network, leading to profound immune dysregulation (Zhang et al., 2025). First, dysbiosis impairs Treg differentiation and reduces their proportion, accompanied by a decrease in SCFA-producing beneficial bacteria, which collectively promotes the excessive release of pro-inflammatory cytokines such as IL-1, IL-6, IL-17, IL-1*β*, and TGF-β (Yassin et al., 2025). These cytokines not only exacerbate intestinal inflammation but also cross the compromised blood–brain barrier (BBB) via systemic circulation, triggering neuroinflammation in the central nervous system (CNS) (Yassin et al., 2025). Second, intestinal accumulation of *α*-synuclein in PD can induce cytotoxic activity in CD8^+^T cells, which may migrate across the disrupted BBB and directly target dopaminergic neurons, accelerating neurodegeneration (Zhang et al., 2025). Furthermore, molecular mimicry in the gut contributes to autoimmune cascades: bacterial amyloid proteins can activate innate immune receptors (e.g., TLR4) or directly induce *α*-synuclein misfolding and aggregation, perpetuating inflammatory burden in the gastrointestinal tract, systemically, and in the CNS, ultimately worsening cognitive decline (Caballero-Villarraso et al., 2025).

Epidemiological evidence provides strong support for the link between immune dysregulation and PD pathogenesis. Individuals with inflammatory bowel disease (IBD)-a condition characterized by chronic gut inflammation, gut microbiota dysbiosis, and elevated TNF-α levels-have a significantly increased risk of developing PD (Liu et al., 2020). Notably, treatment with anti-TNF therapies (the gold standard for IBD management) has been shown to reduce PD risk, providing direct clinical evidence that TNF-α-mediated immune signaling contributes to neurodegeneration in PD (Rafie et al., 2023). This epidemiological data reinforces the critical role of gut-derived immune dysregulation in PD-related cognitive impairment.

Exercise, as an effective non-pharmacological intervention, mitigates neuroinflammation and improves cognitive function through multi-dimensional modulation of the gut microbiota-immune axis (Ajith and Sreejith, 2025). Firstly, exercise enhances gut microbial diversity and enriches SCFA-producing bacteria (Caballero-Villarraso et al., 2025), which activate G protein-coupled receptors (GPR41, GPR43) to regulate immune cell function, promote Treg differentiation, suppress Th17 activation, and reduce pro-inflammatory cytokine release, thereby restoring immune homeostasis (Suda and Matsuda, 2022). Secondly, exercise improves intestinal barrier integrity, reduces permeability, and limits translocation of bacterial products such as LPS into circulation, attenuating peripheral and central immune activation (Zhang et al., 2023). Additionally, exercise directly suppresses NLRP3 inflammasome overactivation, decreasing IL-1β and IL-18 secretion (Chen J. et al., 2025). The gut microbiota acts as a bridge in this process—exercise-induced expansion of anti-inflammatory bacteria modulates immune cell signaling via microbial metabolites, further enhancing neuroprotection (Caballero-Villarraso et al., 2025). Collectively, these mechanisms underscore the value of exercise as a targeted intervention for PD-related cognitive impairment via the gut-brain-immune axis.

#### 4.2.5 The mechanism of improving cognitive ability based on the brain-gut axis: neurotrophic factors and neuroprotective signals

Neurotrophic factors (such as brain-derived neurotrophic factor BDNF and nerve growth factor NGF) play an important role in the brain-gut microbiota axis and are significantly regulated by exercise (Matin and Dadkhah, 2024). The brain-gut microbiota axis is a two-way communication system that integrates neural, immune and metabolic signals. The maintenance of its homeostasis is critical to nervous system health. Intestinal flora dysbiosis not only triggers systemic inflammatory responses, but also leads to neurodegeneration and cognitive dysfunction by destroying the BBB and activating inflammatory pathways in the CNS. In this process, the expression levels of neurotrophic factors such as BDNF are regulated by intestinal flora and their metabolites (such as SCFAs), thereby affecting the survival, repair and synaptic plasticity of neurons (Sanaeifar et al., 2024).

In addition to the indirect regulation of BDNF via intestinal microbiota, recent studies have focused on elucidating the direct interplay between exercise and BDNF, independent of microbial contributions (Rafie et al., 2023). BDNF is a key neurotrophic factor that promotes neuronal survival, synaptic plasticity, and neurogenesis in brain regions critical for cognitive function (e.g., hippocampus, prefrontal cortex) (Ajith and Sreejith, 2025). A study confirmed that exercise-induced BDNF upregulation in the striatum mediates improved motor performance by promoting dopaminergic transmission in aging mice (Rafie et al., 2023), while another study demonstrated that BDNF deficiency exacerbates cognitive decline in PD models by impairing synaptic connectivity in the hippocampus (Ajith & Sreejith, 2025). These findings indicate that the regulatory network among exercise, BDNF, and microbiota is multi-faceted: exercise can upregulate BDNF directly via neurotrophic signaling pathways (e.g., PGC1-*α*/CREB) (Hao et al., 2022) or indirectly via microbiota-derived metabolites (e.g., butyrate) (Białecka-Dębek et al., 2021), while BDNF may in turn modulate gut microbiota composition through the brain-gut axis (Sanaeifar et al., 2024). Clarifying the hierarchical and interactive relationships among these three factors will be a key direction for future research to optimize exercise intervention strategies for PD (Ajith and Sreejith, 2025).

As an effective non-pharmacological intervention, exercise can increase the expression of neurotrophic factors through multiple mechanisms. Studies have shown that exercise promotes the increase in the abundance of beneficial intestinal flora (such as Lactobacillus and Bifidobacterium), enhances intestinal barrier function, and reduces the release of inflammatory factors, thereby indirectly increasing the level of BDNF in the brain and promoting neurological repair and cognitive function improvement (Rafie et al., 2023). For example, in the cerebral ischemia–reperfusion injury model of diabetic rats, exercise improves cognitive function by regulating intestinal flora and increasing SCFAs levels, promoting BDNF expression, reducing oxidative stress and inflammatory responses (Asuku et al., 2024). In addition, exercise also regulates the secretion of FNDC5 by activating the PGC1-*α*/CREB signaling pathway, further promoting the expression of BDNF, forming a positive feedback loop for neuroprotection (Hao et al., 2022).

The signaling between BDNF and its receptor TrkB plays a key role in the occurrence, differentiation, survival and synaptic transmission of neurons. Its insufficient expression is closely related to the pathogenesis of various neuropsychiatric diseases such as depression, Alzheimer’s disease, and PD. The upregulation of neurotrophic factors such as BDNF by exercise is also inseparable from changes in intestinal flora metabolites. SCFAs (such as butyrate) have been confirmed to promote neural development and synaptic plasticity by activating the expression of BDNF (Białecka-Dębek et al., 2021). Therefore, exercise forms the core link of neuroprotective signaling in the brain-gut flora axis by regulating the intestinal microecology and promoting the expression of neurotrophic factors.

## 5 Limitations

This study has several limitations that should be acknowledged. First, bibliometric analysis, as a statistical clustering tool, is inherently retrospective and primarily reflects established research centers and mainstream topics based on existing publications. While it effectively identifies the dominant research landscape, it may fail to capture emerging hotspots and cutting-edge directions that have not yet formed sufficient clustering patterns in the literature. Consequently, this approach may overlook potential future research trends and the latest mechanistic discussions that are still in the early developmental stage.secondly, this study included research data from the Web of Science core ensemble database, and the literature on other databases and other languages were not included, and this study may not be able to include all the content within the field, and there may be a certain amount of bias; thirdly, there was no quality evaluation of the included literature, which may have a certain impact on the bibliometric results;fourthly, the search strategy did not include “genetic parkinsonism” due to the focus on idiopathic PD, which limits the integration of findings from genetic PD models that are valuable for dissecting gut-brain and immune mechanisms.

## 6 Conclusion

This article review summarizes the history and current status of exercise research in the field of cognitive function in Parkinson’ s patients from 1999 to 2024. It can be seen from the research trends that the number of publications in this field has increased in recent years, indicating that this field is currently receiving high attention and developing rapidly. The United States has become the country with more publications, and Eberhard Karls University of Tubingen and Berg, Daniela are the institutions and individuals with more publications. According to the results of bibliometric analysis, the symptoms of cognitive impairment that currently attract more attention are mainly mild cognitive impairment, dementia, and depression. The most studied form of exercise is aerobic exercise. The current research focus on the mechanism of action is to explore the impact of exercise on cognitive function from the perspective of the brain-gut axis.

The central role of the brain-gut microbiota axis in the pathogenesis of cognitive impairment in PD has been supported by increasing experimental evidence. As an important bridge connecting the intestines and the CNS, the intestinal flora significantly affects neurological function and cognitive performance by regulating inflammatory responses and metabolic products. Current research shows that intestinal flora imbalance not only aggravates neuroinflammation and oxidative stress, but also interferes with the homeostasis of the brain environment through multiple signaling pathways, promoting the gradual deterioration of cognitive function. However, different studies still have certain differences in the specific composition changes of bacterial flora and the details of their pathogenic mechanisms, which suggests that we need more refined and multi-level analysis methods to clarify the complex bacterial flora-neural interaction network.

As a safe and low-cost non-pharmacological intervention, exercise has become increasingly valuable in the management of cognitive impairment in PD. A large number of clinical and basic studies have shown that regular exercise can not only optimize the diversity and function of intestinal flora, but also activate the expression of neurotrophic factors such as BDNF, reduce neuroinflammation and oxidative damage, thereby promoting the improvement of cognitive function. These biological effects mediated by exercise provide new perspectives and pathways for understanding the intervention of cognitive impairment in PD. At the same time, there are individual differences in the effects of exercise intervention in different patients. How to formulate personalized exercise prescriptions based on the patient’s specific pathological state and living habits is an important direction for future research and clinical application.

## Data Availability

The original contributions presented in the study are included in the article/supplementary material, further inquiries can be directed to the corresponding author/s.
